# Applicability of the DPRA on mixture testing: challenges and opportunities

**DOI:** 10.1007/s00204-023-03551-y

**Published:** 2023-07-07

**Authors:** Quinten Marcelis, Eric Deconinck, Vera Rogiers, Tamara Vanhaecke, Bart Desmedt

**Affiliations:** 1 In Vitro Toxicology and Dermato-Cosmetology (IVTD), VUB, Laarbeeklaan 103, 1090 Jette, Belgium; 2 Medicines and Medicinal Health Products, Sciensano, Juliette Wytsmanstraat 14, 1050 Elsene, Belgium

**Keywords:** Skin sensitization, DPRA, In chemico, Mixture toxicology

## Abstract

The in chemico direct peptide reactivity assay (DPRA) is validated to assess protein reactivity of chemical compounds, relating to the molecular initiating event of skin sensitization induction. According to OECD TG 442C, the DPRA is technically applicable to test multi-constituent substances and mixtures of known composition, even though limited experimental data are publicly available. First, we assessed the DPRA’s predictive capability for individual substances, but at concentrations other than the recommended 100 mM, i.e., based on the LLNA EC3 concentration (Experiment A). Next, the applicability of the DPRA to test unknown mixtures was assessed (Experiment B). Here, the complexity of unknown mixtures was reduced to mixtures containing either two known skin sensitizers with varying potencies, or a combination of a skin sensitizer with a non-skin sensitizer, or multiple non-sensitizers. Experiments A and B revealed that one extremely potent sensitizer (oxazolone) was incorrectly classified as a non-sensitizer when tested at its low EC3 concentration of 0.4 mM instead of the suggested molar excess conditions of 100 mM (Experiments A). For binary mixtures tested in experiments B, the DPRA was able to distinguish all skin sensitizers and the strongest skin sensitizer in the mixture was determinant for the overall peptide depletion of a sensitizer. In conclusion, we confirmed that the DPRA test method can be used efficiently for well-known characterized mixtures. However, when deviating from the recommended testing concentration of 100 mM, caution should be taken in case of negative results, limiting the DPRA’s applicability for mixtures of unknown composition.

**Supplementary Information:**

The online version contains supplementary material available at 10.1007/s00204-023-03551-y.

## Introduction

Allergic contact dermatitis (ACD) continues to be an important adverse reaction, with approximately 15–20% of the general population becoming sensitized during their lifetime (Peiser et al. 2012). Many consumer products contain substances that can cause an allergic reaction, such as nickel present in jewelry or fragrances, and preservatives present in consumer products (Peiser et al. 2012). Yet, to safeguard the consumer’s health, a regulatory framework has been introduced by the EU where (i) raw material suppliers must identify and convey any sensitizing concerns (European Commission), as required by the Registration, Evaluation, Authorization and Restriction of Chemicals Regulation (REACH regulation) (1) (European Commission 2006), and (ii) finished products entering the EU market must comply with the General Product Safety Directive (GPSD). The latter mandates that every product placed on the EU market shall be safe (European Commission 2001). As a result, the manufacturer bears the burden of proof that their consumer products are safe before being placed on the market. Furthermore, (iii) hazardous chemicals are restricted in concentration and applicability domain, e.g., in cosmetic or children’s toys (European Commission 2009, 2019) and (iv) labeling of 26 well-known allergens is mandatory for cosmetics when 10 ppm is exceeded in leave-on products and 100 ppm in rinse-off products. In general, there is a need for a comprehensive risk assessment, taking into account the intended exposure scenario, the hazard characterization of its raw materials and additives.

Increasing insight in the immunological process of ACD resulted in an Adverse Outcome Pathway (AOP) for sensitization and the determination of four immunological key events (KEs) involved in the development of skin sensitization. These four KEs are covalent binding of allergens to skin proteins (haptenation) (KE1 or MIE, Molecular Initiating Event), the subsequent activation of keratinocytes (KE2), activation of dendritic cells (KE3) and the proliferation of T-lymphocytes (KE4) (OECD 442C) (2014). Driven by technological improvements and ethical considerations in line with the 3Rs principle of Replacement, Reduction and Refinement of animal testing, the OECD has approved numerous non-animal methods addressing the first three KEs of the skin sensitization AOP. These include for KE1: the Direct Peptide Reactivity Assay (DPRA), the Amino acid Derivative Reactivity Assay (ADRA) and kinetic Direct Peptide Reactivity Assay (kDPRA) (OECD TG 442C) (2021b); for KE2: the ARE-Nrf2 Luciferase Test Method (KeratinoSens™ and LuSens, OECD TG 442D) (2018a); for KE3: the human Cell Line Activation Test (h-CLAT), U937 cell line activation Test (U-SENS™) and Interleukin-8 Reporter Gene Assay (IL-8 Luc assay), Genomic Allergen Rapid Detection (GARD™) for the detection of skin sensitization (GARDskin™) (OECD TG 442E) (2018b).

These validated in chemico and in vitro tests are implemented in the EU regulatory framework to meet the paradigm shift towards minimizing animal experimentation. In this context, animal testing of finished cosmetic products and cosmetic ingredients is already fully prohibited in the EU (Regulation (EC) No 1223/2009), whereas the REACH regulation favors in chemico and in vitro techniques for skin sensitization testing and authorizes the use of the Murine Local Lymph Node Assay (LLNA) only if the chemical is not suitable for non-animal tests (Regulation (EC) No 1907/2006).

In particular, the DPRA focuses on the molecular initiating event (KE1), namely the formation of a hapten–protein complex, which is a prerequisite in developing ACD. The test method assumes that most chemical allergens are small molecules with electrophilic properties, which react with electron-rich groups of nucleophilic amino acids of skin proteins and can, therefore, covalently bind them (Chipinda et al. 2011). This covalent binding is simulated in chemico by quantifying the reactivity of possible sensitizers towards the amino acids cysteine and lysine present in synthetic peptides. Next to its short analysis time and easy instrumental setup, the DPRA offers a great sensitization prediction accuracy of 89% when compared to the LLNA (Gerberick et al. 2007). One drawback, however, is that the DPRA, like the other mentioned non-animal test methods, is not recommended as a stand-alone test for skin sensitization hazard identification. To circumvent this problem, the DPRA is incorporated into an integrated testing strategy and defined approaches, where multiple information sources (in silico, in chemico*, *in vitro and historical in vivo data) are combined to perform a correct hazard assessment (OECD 2021b).

With increasing regulatory acceptance and adoption of non-animal methodologies for evaluating skin sensitization, new challenges have emerged. Indeed, the aforementioned test methods were validated using pure test substances and hence their applicability domain only focuses on testing single chemicals. This is in sharp contrast to the testing requirements for various industrial sectors dealing with complex mixtures such as essential oils in the cosmetics industry or medical device extracts to be evaluated for biocompatibility.

**Fig. 1:**
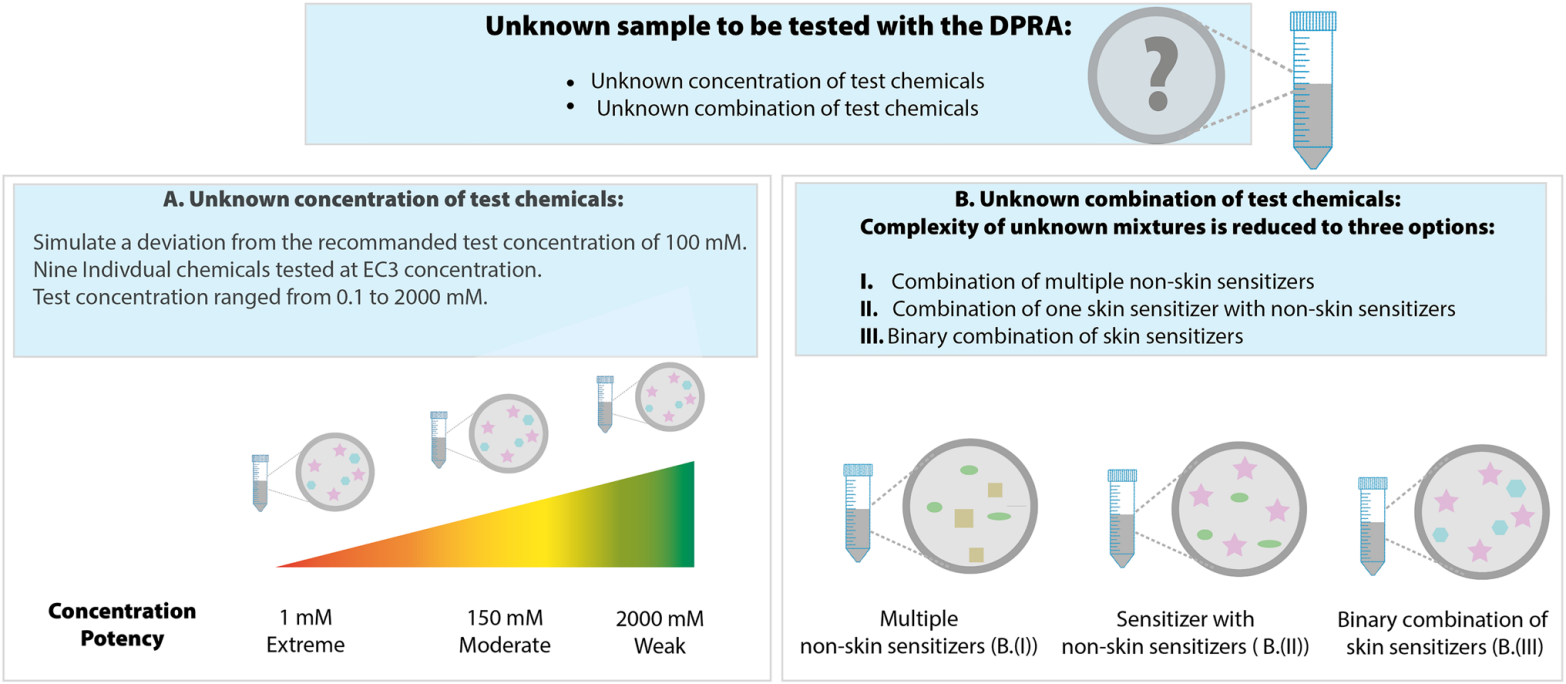
Graphical overview of the followed methodological approach

Expanding the DPRA’s applicability domain to unknown mixtures could be helpful in resolving the latter concerns. In this context, two sets of experiments (see experiments A and B in Fig. 1) are conducted in this study to investigate the plausible applicability or drawbacks of the DPRA in case of a sample with either an ingredient with unknown concentration or an unknown combination of ingredients (Fig. 1). The first set of experiments focused on the most basic representation of an ‘unknown sample’, namely a solution containing a known substance, but at an unknown concentration (Experiment A). Test chemicals were tested at their respective EC3 concentrations, which ranged from 0.1 mM to 2000 mM, rather than the OECD-recommended fixed concentration of 100 mM. This means that the more potent a sensitizer, the lower its DPRA testing concentration will be. Next, various combinations of chemical mixtures were tested (Experiment B). Here, the complexity of an unknown mixture was reduced to a combination of (i) non-skin sensitizers, (ii) a skin sensitizer in combination with a non-skin sensitizer (iii) and two skin sensitizers. The test chemicals under investigation covered a wide range of skin-sensitizing potencies to represent plausible chemical mixtures.

## Materials and methods

### Chemicals and reagents

Experiments were performed with eight technical proficiency chemicals mentioned in OECD TG 442C and four common fragrance allergens. A complete overview of the chemicals with corresponding CAS-number and skin sensitization potency is given in Appendix A. Synthetic peptides, cysteine and lysine, were obtained from Thermo Fischer (Massachusetts, USA). Buffer solutions for both peptides contained disodium phosphate (CAS n° 7558-79-4) or ammonium acetate (CAS n° 631-61-8), obtained from Merck (Darmstadt, Germany).

### DPRA procedure

The performance of the DPRA followed the standard operating procedure as mentioned in OECD TG 442C (2021b). Stock solutions of cysteine and lysine were prepared at 0.667 mM in a phosphate buffer (pH 7.5) or an ammonium acetate buffer (pH 10.2), respectively. Cysteine and lysine peptide solutions were incubated with the test chemicals in glass vials at a 1:10 (for cysteine) or 1:50 ratio (for lysine). To 750 µl of cysteine peptide solution, 250 µl of test chemical was added in each vial. To 750 µl of lysine peptide solution, 50 µl of test chemical and 200 µl acetonitrile were added to each vial. The samples were then incubated at 25 °C for 24 h in the dark in an Eppendorf ThermoMixer™ (Hamburg, Germany), prior to HPLC analysis.

In addition, reference controls that only contained the peptide solution and dissolution solvent were included to verify system suitability, examine protein stability and to confirm that the solvent (acetonitrile) did not negatively affect peptide depletion. Cinnamic aldehyde, functioning as the positive control, was prepared in acetonitrile at a concentration of 100 mM. Furthermore, a calibration curve was prepared for each peptide containing 20% acetonitrile and 80% buffer solution. Using serial dilution of the peptide stock solutions, six calibration solutions encompassing the range of 0.534–0.0167 mM were produced. The standard calibration curves were considered linear when *r*^2^ was greater than 0.99.

Sample analysis was performed on a Waters Alliance™ HPLC System (Milford, MA, USA), equipped with a C18 reverse-phase column (Zorbax SB-C18; 2.1 × 100 mm) from Agilent Technologies (Santa Clara, California, USA) and coupled with a UV detector operated at 220 nm. 7 μL of each sample was injected into the HPLC system; injection was carried out twice. The entire system was equilibrated at 30 °C with 50% phase A (0.1% (v/v) trifluoroacetic acid in water) and 50% phase B (0.085% (v/v) trifluoroacetic acid in acetonitrile) for at least 2 h before analysis. The HPLC analysis was conducted at a flow rate of 0.35 ml/min and with a linear gradient from 10 to 25% acetonitrile over 10 min, followed by a rise to 90% acetonitrile to rinse the column.

The percent peptide depletion was determined based on the reduction of cysteine and lysine concentrations in the samples compared to the concentration observed in the reference controls (Eq. 1). The criterion to distinguish sensitizers from non-sensitizers was set at 6.38% mean depletion for the cysteine and lysine prediction model, and 13.89% mean depletion for the cysteine prediction model. Mean activities were calculated from two independent experiments conducted in duplicate:

1 $${\text{Percent }}\,{\text{peptide }}\,{\text{depletion}} = \left[ {1 - \left( {\frac{{\text{Peptide peak area in replicate injection}}}{{\text{Peptide peak area in reference controls C}}}} \right)} \right] \times 100$$

### Methodology

A. Unknown concentration of test chemicals.

Nine test chemicals with varying sensitization potencies were selected and test concentrations were prepared based upon their LLNA EC3 concentrations ranging from 0.46 to almost 2000 mM. This test set comprised several technical proficiency compounds and common fragrances used in the cosmetics industry (Table. 1). Experiments were performed in parallel using cysteine and lysine peptides and carried out in duplicate to confirm reproducibility. Results obtained with the lysine peptides are shown in Appendix B.

**Table 1:** Overview of EC3 concentration in % and mM of the tested components obtained from the literature (Loveless et al. 2010)

Compound	Potency classification	EC_3_ value (m/m %)	EC_3_ value (mM)
Oxazolone	Extreme	0.01	0.46
2,4-Dinitrochlorobenzene	Extreme	0.08	3.95
Formaldehyde	Strong	0.4	133.20
Cinnamic aldehyde	Moderate	2.0	151.33
Citral	Weak	5.7	374.41
Eugenol	Weak	13	791.72
Linalool	Weak	30	1944.90

Included non-sensitizers (6-methylcoumarin and lactic acid) have been tested at 30% m/m

B. Unknown combination of test chemicals.
  (i) Combination of non-sensitizers.
  We tested a “surrogate mixture” containing non-sensitizers commonly found in a variety of products (e.g., consumer products and cosmetics). The selected compounds present no skin sensitization properties as confirmed by available in vivo data. The eight selected compounds were 1-butanol, 6-methylcoumarin, lactic acid, 4-methoxyacetophenone, glycerol, benzyl alcohol, dimethyl isophthalate and propyl paraben. The final concentration of each individual test chemical in the surrogate mixture was 100 mM. Experiments with lysine peptide are performed in parallel and results will be shown in Appendix B.
  (ii) Combination of skin sensitizer and non-skin sensitizers.
  The surrogate mixture containing the same eight non-sensitizers was tested together with one sensitizer for each skin-sensitizing potency class: 2,4-dinitrochlorobenzene (extreme), formaldehyde (strong), benzylideneacetone (moderate) and farnesal (weak). The introduced chemicals were all present in a final concentration of 100 mM, as suggested by the DPRA protocol for testing single compounds. Depletion values of the individual skin sensitizer and the individual skin sensitizer present in the surrogate mixture were compared to detect deviations in the peptide depletion caused by mixture effects. Experiments with lysine peptide are performed in parallel and results are shown in Appendix B.
  (iii) Combination of skin sensitizers.

16 binary mixtures containing either two sensitizers with varying sensitization potencies, a combination of a skin sensitizer with a non-skin sensitizer or two non-sensitizers, were tested at 100 mM per compound in duplicate (Table 2). Consequently, the created binary mixtures comprised each combination of two potency classes. The constituents of those binary mixtures were the technical proficiency compounds mentioned in OECD guideline TG 442C.

**Table 2:** Binary mixtures from eight technical proficiency compounds mentioned in OECD TG 442C

Substance (potency)	2,4-Dinitrochlorobenzene (extreme)	Formaldehyde (strong)	Farnesal (weak)	1-Butanol (non-sensitizer)
Oxazolone (extreme)	1	2	3	4
Benzylideneacetone (moderate)	5	6	7	8
2,3-Butanedione (weak)	9	10	11	12
Lactic acid (non-sensitizer)	13	14	15	16

Each binary mixture, created by mixing a substance from the upper row with one of the substances from the first column, is given a number. The respective skin sensitization potency of each compound is indicated in between brackets (2021)

## Results

### Unknown concentration of test chemicals

Table 3 presents the predictions of the DPRA obtained for nine sensitizing compounds (2,4-dinitrochlorobenzene, 6-methylcoumarin, cinnamaldehyde, citral, eugenol, formaldehyde, lactic acid, linalool, oxazolone) when using their EC3 values instead of the recommended testing concentration of 100 mM. A comparison between the classification by the DPRA when the chemical is tested at its EC3 concentration and the obtained LLNA classification resulted in the correct classification of six out of the seven skin sensitizers (linalool, eugenol, cinnamaldehyde, 2–4-dinitrochlorobenzene, formaldehyde, and citral). In addition, a correct classification for two non-sensitizers, namely lactic acid and 6-methylcoumarin, is also obtained. The incorrectly classified chemical, i.e., the extreme sensitizer oxazolone, resulted in a cysteine depletion of only 6.90% at its EC3 concentration of 0.46 mM, in comparison to a cysteine depletion of 71.5% when tested at the 200 times higher mandatory testing concentration of 100 mM.

**Table 3:** Comparison of the outcome by DPRA at EC3 concentration using the cysteine prediction model versus the LLNA classification

Compound	Outcome by DPRA	Classification by LLNA
Oxazolone	Non-sensitizer	Sensitizer
2,4-Dinitrochlorobenzene	Sensitizer	Sensitizer
Formaldehyde	Sensitizer	Sensitizer
Cinnamic aldehyde	Sensitizer	Sensitizer
Citral	Sensitizer	Sensitizer
Eugenol	Sensitizer	Sensitizer
Linalool	Sensitizer	Sensitizer
6-Methylcoumarin	Non-sensitizer	Non-sensitizer
Lactic acid	Non-sensitizer	Non-sensitizer

Nine compounds were evaluated for their skin-sensitizing potential according to the reference method LLNA and the two prediction models of the DPRA

### Unknown combination of test chemicals

#### Combination of non-sensitizers

**Fig. 2:**
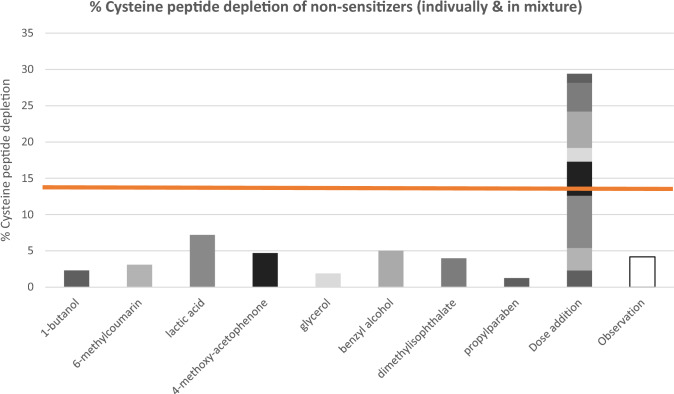
Peptide reactivity measured for each individual non-skin sensitizer, the hypothetical response and the observed response of the surrogate mixture at 100 mM using the cysteine prediction model. Reactivity of the mixture and individual test chemicals (gray) are expressed by their mean percent peptide depletion of cysteine. Observed reactivity of the mixture (white). The orange line indicates the discrimination between skin sensitizers and non-skin sensitizers (14%)

The mixture comprising eight different non-sensitizers (1-butanol, 6-methylcoumarin, lactic acid, 4-methoxyacetophenone, glycerol, benzyl alcohol, dimethyl isophthalate, and propylparaben) was classified as not reactive towards cysteine-containing peptides (< 14% cysteine peptide depletion) (Fig. 2). As an illustration, the mixture’s expected peptide depletion is shown for a scenario where dose additions would apply. This resulted in over 30% cysteine peptide depletion and was, therefore, incorrectly classified as a skin-sensitizing mixture (> 14% cysteine peptide depletion).

#### Combination of a skin sensitizer with non-skin sensitizers

**Fig. 3:**
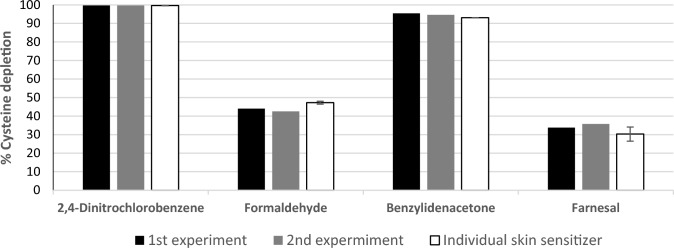
Reactivity measured for the pseudo-binary mixtures containing one skin sensitizer at 100 mM using the cysteine prediction model. Reactivity of the pseudo-binary mixtures and individual test chemicals (white) are expressed by their mean percent peptide depletion of cysteine. Data obtained from two independent mixture experiments are represented in black and gray, being the 1st and 2nd experiments, respectively

Four pseudo-binary mixtures containing the same eight non-sensitizers as above plus one additional sensitizer were tested. As shown in Fig. 3, similar peptide depletion values using the cysteine prediction model were observed for the individual sensitizers and when tested in the surrogate mixture, indicating correct classification.

#### Combination of skin sensitizers

Table 4 provides an overview of the results of the 16 binary mixtures of proficiency compounds tested with the DPRA. Similar to individual compounds, we found a positive correlation between the presence of skin sensitizers in the mixtures and protein depletion. No misclassification was observed when using both the cysteine and cysteine-lysine prediction models, resulting in excellent accuracy for the tested binary mixtures. All 15 mixtures that contained at least one substance that is individually classified as a skin sensitizer according to LLNA data were found to be positive. In addition, the mixture containing the two non-sensitizers (mixture 16: 1-butanol and lactic acid) was correctly predicted negative for skin sensitization. Overall, these results suggest that the DPRA can distinguish skin sensitizers from non-sensitizers when testing a binary mixture at 100 mM per compound.

**Table 4:** Reactivity expressed as mean percent cysteine peptide depletion obtained for the 16 binary mixtures tested (100 mM/100 mM)

Substance (potency)	2,4-Dinitrochlorobenzene (extreme)	Formaldehyde (strong)	Farnesal (weak)	1-Butanol (non-sensitizer)
Oxazolone (extreme)	93.7	77.8	65.9	73.9
Benzylideneacetone (moderate)	99.8	88.4	96.0	93.7
2,3-Butanedione (weak)	99.5	87.0	81.9	79.3
Lactic acid (non-sensitizer)	99.9	46.6	37.3	0

**Fig. 4:**
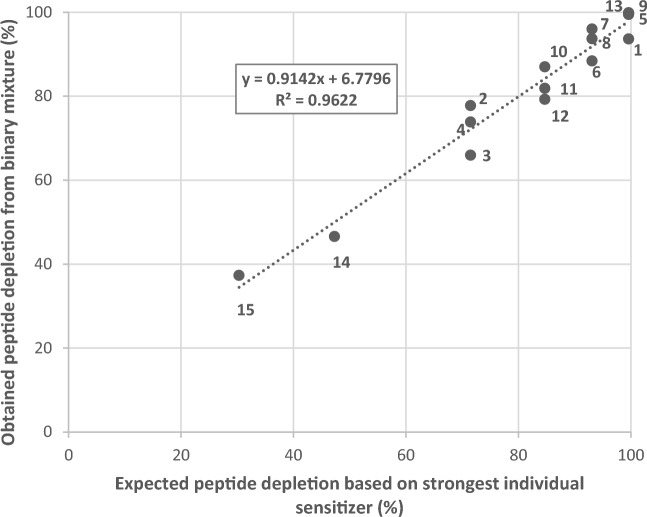
Response modulation of the expected peptide depletion based on the strongest individual sensitizer and its mixture using cysteine-containing peptides. Numbers shown correspond with the binary mixtures, as defined in Table 2

A comparison of the protein depletion values obtained for the binary mixtures and the value of the individual components suggests that the overall reactivity of the mixture is driven by its strongest sensitizing component. To further illustrate this hypothesis, the depletion percentage of the binary mixture (y-axis) was plotted against the depletion percentage of the strongest individual sensitizer (x-axis) (Fig. 4). Based on the 16 binary mixtures tested, a linear relationship is observed with a correlation coefficient of 0.96, clearly indicating a positive association between peptide depletion of the strongest sensitizer present in the mixture and the overall mixture peptide depletion.

## Discussion

Nine compounds were evaluated with the DPRA at diverging testing concentrations, i.e., at their EC3 concentration. This experimental setup significantly deviates from the OECD testing guideline, which stipulates that chemicals must be tested at a fixed concentration of 100 mM. Yet, a correct classification was obtained for eight of the nine compounds. Only the extremely potent skin sensitizer oxazolone, which had the lowest tested EC3 concentration (0.46 mM), gave a false-negative result. This could be due to the extremely low concentration tested where no molar excess of the test chemical was present. It is, therefore, indicated that some extreme sensitizers, present at low concentration in an unknown mixture would not trigger measurable peptide depletion. Yet, another extreme sensitizer, 2,4-dinitrochlorobenzene, was tested at 4 mM and was correctly classified, showing that deviating from the 100 mM can in some cases still be effectively predicted by the DPRA. The initial insight generated by this preliminary investigation helped to generate the hypothesis that a thorough sensitivity analysis per individual compound should be investigated, i.e., testing several concentrations below 100 mM to address the sensitivity of the DPRA. In addition, these results should always be confirmed by a second in vitro test as required by the 2 out of 3 approach set out in OECD TG 497 (OECD 2021b).

Although the DPRA is technically applicable to analyze mixtures of known compositions, the literature on this subject is limited. Our second set of experiments, focusing on mixtures with known composition, showed that the DPRA classified combinations of skin sensitizers or non-sensitizers with great accuracy. From our results, it is also clear that the strongest sensitizing component present in the mixture drives the reactivity in the DPRA. Likewise, no inhibitory or masking effect was observed upon introducing a second chemical. Although with a smaller dataset of only two binary mixtures of fragrance aldehydes, i.e., hydroxycitronellal-citral and citral-cinnamaldehyde, similar observations were done by Lang et al*.* (2017). Hence, we were able to confirm this finding and extend the data to a set of 16 combinations of sensitizers with varying potency and industrial use (e.g., fragrances and additives).

In addition, there was no significant difference in peptide depletion when a skin sensitizer was tested individually or in a mixture with multiple non-sensitizers. This means that non-skin-sensitizing chemicals have no additive or masking effect. These findings are consistent with in vivo experiments using LLNA, which show that skin sensitizers included in essential oils evoked immunogenic responses similar to the pure component (Lalko and Api 2006). Likewise, when non-sensitizing plant extracts, spiked with different doses of common fragrance allergens, were tested in the in vitro KeratinoSens assay, no general masking effect was observed (Andres et al. 2013). As expected, it seems that the DPRA proved to be equally capable of distinguishing skin sensitizers from non-sensitizers in mixtures with a sensitizer present at 100 mM.

The counterpart of the DPRA, the amino acid derivative reactivity assay (ADRA), has been subject to similar mixture testing (Yamamoto et al. 2019). As in our study, non-sensitizers present in a mixture did not alter the discrimination between skin sensitizers and non-sensitizers. Interestingly, a tenfold deviation from the mandatory test concentration, resulted in a comparable accuracy for the classification of skin sensitizers. Therefore, it was concluded that, although further validation is necessary, test chemicals with unknown molecular weights can be tested with the same predictive capacity as the conventional ADRA test preparations. Albeit a different methodology, we observed a potential limitation from the DPRA as well when extreme diluted skin sensitizers are tested. In addition to the challenges faced by the in vitro tests, it is important to acknowledge that in vivo tests, i.e., LLNA, encounter similar limitations. Upon establishing the dose–response curve (and the EC3%), it can be observed that for each skin sensitizer, if present at a low enough concentration, no significant induction of proliferative response will occur (OECD 2010), which is similar to what is observed in our experiments with oxazolone tested at 0.46 mM, a skin sensitizer at a low concentration, yielding no significant peptide depletion.

With this knowledge, especially from our first set of experiments, DPRA results obtained from unknown mixtures should be interpreted with caution. Recent literature on the safety testing of medical devices and consumer goods already revealed discrepancies on this matter (Svobodova et al. 2020; Svobodová et al. 2021). Medical device extracts were tested with the DPRA, but did not always give the same classification when using in vivo (LLNA) or in vitro methodologies (KeratinoSens). It could be that possible sensitizers were extremely diluted due to the extraction conditions. Taken together, our findings provide some support for the conceptual premise that a negative result obtained with DPRA for an (unknown) mixture should be interpreted with caution, due to the possibility that extreme sensitizing compounds could be present at very low concentrations. This also emphasizes the importance of chemical characterization of mixtures prior to in vitro testing, e.g., DPRA, to ensure compatibility with the test’s applicability domain and perform compound specific sensitivity analyses with the DPRA.

However, since in our three sets of experiments no false positives were observed, a positive result obtained from a mixture could be seen as a true positive and should be further investigated in accordance with the AOP using different in vitro tests, in line with the general recommendation from OECD TG 497 promoting a defined approach of two or more in vitro methodologies to assess skin sensitization (OECD 2021a).

## Conclusion

The goal of this study was to investigate the potential challenges the DPRA faces when unknown mixture samples would be tested. We observed that a potential limitation lies in the fact that extremely low concentrations of skin sensitizers present in a mixture could possibly trigger false-negative results. Furthermore, we could confirm that a mixture of known composition can be tested by the DPRA at the predetermined concentration of 100 mM (OECD TG 442 C). Our findings suggest that the joint action of two sensitizing chemicals in a mixture is not larger than the effect of the strongest sensitizer.

## Supplementary Information

Below is the link to the electronic supplementary material.

Supplementary file1 (DOCX 55 KB)

## Data Availability

Data is available through the supplementary file.
