# Supplementary material for: Applicability of the DPRA on mixture testing: challenges and opportunities
Source: Arch Toxicol. 2023 Jul 7;97(9):2453–61. doi: 10.1007/s00204-023-03551-y (PMC10404183; doi:10.1007/s00204-023-03551-y)
Supplement: Supplementary file 1 — Supplementary file1 (DOCX 55 KB) [file 204_2023_3551_MOESM1_ESM.docx]

# Appendix A

| Name | CASRN | *In vivo*  prediction | Reason for inclusion |
| --- | --- | --- | --- |
| 2,4-Dinitrochlorobenzene | 97-00-7 | Sensitizer  (extreme) | Technical proficiency compound DPRA |
| Oxazolone | 15646-46-5 | Sensitizer  (extreme) | Technical proficiency compound DPRA |
| Formaldehyde | 50-00-0 | Sensitizer  (strong) | Technical proficiency compound DPRA |
| Benzylideneacetone | 122-57-6 | Sensitizer  (moderate) | Technical proficiency compound DPRA |
| Farnesal | 19317-11-4 | Sensitizer  (weak) | Technical proficiency compound DPRA |
| 2,3-Butanedione | 431-03-8 | Sensitizer  (weak) | Technical proficiency compound DPRA |
| 1-Butanol | 71-36-3 | Non-sensitizer | Technical proficiency compound DPRA |
| Lactic Acid | 50-21-5 | Non-sensitizer | Technical proficiency compound DPRA |
| Linalool | 78-70-6 | Sensitizer  (weak) | Cosmetic ingredient |
| Eugenol | 97-53-0 | Sensitizer  (weak) | Cosmetic ingredient |
| Citral | 5392-40-5 | Sensitizer (Moderate) | Cosmetic ingredient |
| Cinnamic aldehyde | 104-55-2 | Sensitizer (Moderate) | Positive control in DPRA |
| 6-methylcoumarin | 92-48-8 | Non-sensitizer | Technical proficiency compound DPRA |
| 4-methoxyacetophenone | 100-06-1 | Non-sensitizer | Technical proficiency compound DPRA |
| glycerol | 56-81-5 | Non-sensitizer | Technical proficiency compound ADRA |
| benzyl alcohol | 100-51-6 | Non-sensitizer | Technical proficiency compound ADRA |
| dimethyl isophtalate | 1459-93-4 | Nonsensitizer | Technical proficiency compound ADRA |
| propyl paraben | 94-13-3 | Nonsensitizer | Technical proficiency compound ADRA |

The selection of technical proficiency compounds was as follows: 2,4-dinitrochlorobenzene (CAS n° 97-00-7), oxazolone (CAS n° 15646-46-5), formaldehyde (CAS n° 50-00-0, benzylideneacetone (CAS n° 122-57-6), farnesal (CAS n° 19317-11-4), 2,3-butanedione (CAS n° 431-03-8), 1-butanol (CAS n° 71-36-3), lactic acid (CAS n° 50-21-5). Additionally, four common fragrance allergens have been selected that are frequently incorporated in cosmetics. Linalool (CAS n° 78-70-6), eugenol (CAS n° 97-53-0), citral (CAS n° 5392-40-5) and cinnamic aldehyde (CAS n° 104-55-2). The compound selection aimed to be chemically diverse by the inclusion of a wide range of skin sensitization potency categories (2 extreme-, 1 strong-, 2 moderate-, 5 weak- and 2 non-sensitizers).

Additionally selected non-sensitizers to incorporate in a surrogate-mixture were 6-methylcoumarin (CAS n° 92-48-8) , 4-methoxyacetophenone (CAS n° 100-06-1), glycerol (CAS n° 56-81-5), benzyl alcohol (CAS n° 100-51-6), dimethyl isophtalate (CAS n° 1459-93-4) and propyl paraben (CAS n° 94-13-3). Furthermore, cinnamic aldehyde (CAS n° 104-55-2) functioned as positive control during the DPRA, as mentioned by OECD TG 442C. All chemicals were obtained from Thermo Fischer (Massachusetts, USA).

# Appendix B - Cysteine & Lysine prediction model

Comparison of the outcome by DPRA at EC3 concentration using the cysteine & lysine prediction model *versus* the LLNA classification. Nine compounds were evaluated for their skin sensitizing potential according to the reference method LLNA and the cysteine & lysine prediction model of the DPRA.

| **Compound** | **Outcome by DPRA** | **Classification by LLNA** |
| --- | --- | --- |
| Oxazolone | Non-sensitizer | Sensitizer |
| 2,4-Dinitrochlorobenzene | Sensitizer | Sensitizer |
| Formaldehyde | Sensitizer | Sensitizer |
| Cinnamic aldehyde | Sensitizer | Sensitizer |
| Citral | Sensitizer | Sensitizer |
| Eugenol | Sensitizer | Sensitizer |
| Linalool | Sensitizer | Sensitizer |
| 6-Methylcoumarin | Non-sensitizer | Non-sensitizer |
| Lactic acid | Non-sensitizer | Non-sensitizer |

**Reactivity expressed as mean percent cysteine & lysine peptide depletion obtained for the 16 binary mixtures tested (100mM/100mM).**

| **Substance**  (potency) | **2,4-Dinitrochlorobenzene**  (Extreme) | **Formaldehyde**  (Strong) | **Farnesal**  (Weak) | **1-Butanol**  (Non-sensitizer) |
| --- | --- | --- | --- | --- |
| **Oxazolone**  (Extreme) | 76.04 | 66.67 | 59.96 | 62.34 |
| **Benzylidenacetone**  (Moderate) | 60.94 | 50.04 | 53.34 | 52.87 |
| **2,3-Butanedione**  (Weak) | 77.34 | 89.52 | 61.12 | 59.73 |
| **Lactic acid**  (Non-sensitizer) | 63.23 | 24. | 20.46 | 0.86 |

Reactivity measured for the pseudo-binary mixtures containing one skin sensitizer at 100 mM using the cysteine & lysine prediction model. Reactivity of the pseudo-binary mixtures and individual test chemicals (white) are expressed by their mean percent peptide depletion of cysteine & lysine.

Data obtained from two independent mixture experiments are represented in black and gray, being the 1^st^ and 2^nd^ experiment, respectively.

# Appendix C: Reactivity of the binary mixtures using cysteine prediction model

**Reactivity of the 16 binary mixtures (100mM/100mM) and (50mM/50mM) with results expressed as mean of percent peptide depletion of cysteine.**

|  | | **Cysteine prediction model** | |
| --- | --- | --- | --- |
|  |  | **Mixture (100mM/100mM)** | **Mixture (50mM/50mM)** |
|  |  | Mean of cysteine depletion (%) | Mean of cysteine depletion (%) |
| **1** | 2,4-dinitrochlorobenzene + Oxazolone | 93,65 | 95,30 |
| **2** | Formaldehyde + Oxazolone | 77,77 | 65,45 |
| **3** | Farnesal + Oxazolone | 65,94 | 64,93 |
| **4** | 1-butanol + Oxazolone | 73,86 | 68,65 |
| **5** | 2,4-dinitrochlorobenzene + Benzylidenacectone | 99,79 | 99,45 |
| **6** | Formaldehyde + Benzylidenacetone | 88,40 | 82,87 |
| **7** | Farnesal + Benzylidenacetone | 96,00 | 91,15 |
| **8** | 1-butanol + Benzylidenacetone | 93,70 | 85,66 |
| **9** | 2,4-dinitrochlorobenzene +  2,3-butanedione | 99,47 | 99,30 |
| **10** | Formaldehyde + 2,3-butanedione | 87,01 | 86,04 |
| **11** | Farnesal + 2,3-butanedione | 81,89 | 88,81 |
| **12** | 1-butanol + 2,3-butanedione | 79,25 | 80,58 |
| **13** | 2,4-dinitrochlorobenzene + Lactic acid | 99,89 | 99,82 |
| **14** | Formaldehyde + Lactic acid | 46,59 | 41,27 |
| **15** | Farnesal + Lactic acid | 37,34 | 37,46 |
| **16** | 1-butanol + Lactic acid | 0 | 0 |
